# Pulmonary Protein Oxidation and Oxidative Stress Modulation by *Lemna minor* L. in Progressive Bleomycin-Induced Idiopathic Pulmonary Fibrosis

**DOI:** 10.3390/antiox11030523

**Published:** 2022-03-08

**Authors:** Yanka Karamalakova, Ivaylo Stefanov, Ekaterina Georgieva, Galina Nikolova

**Affiliations:** 1 Department of Medical Chemistry and Biochemistry, Medical Faculty, Trakia University, 11 Armeiska Str., 6000 Stara Zagora, Bulgaria; yanka.karamalakova@trakia-uni.bg (Y.K.); ekaterina.georgieva@trakia-uni.bg (E.G.); 2 Department of Anatomy, Medical Faculty, Trakia University, 11 Armeiska Str., 6000 Stara Zagora, Bulgaria; ivaylo.stefanov@trakia-uni.bg

**Keywords:** bleomycin, idiopathic pulmonary fibrosis, *L. minor*, protein carbonyl content, oxidative stress, antioxidant enzymatic system, antioxidant non-enzymatic system

## Abstract

Bleomycin (BLM) administration is associated with multifunctional proteins inflammations and induction of idiopathic pulmonary fibrosis (IPF). *Lemna minor* L. extract, a free-floating monocot macrophyte possesses antioxidant and anti-inflammatory potential. The aim of the study was to examine the protective effect of *L. minor* extract on lung protein oxidation and oxidative stress modulation by BLM-induced pulmonary fibrosis in Balb/c mice. For this purpose, the protein carbonyl content, advanced glycation end product, nitroxide protein oxidation (5-MSL), and lipid peroxidation (as MDA and ROS), in lung cells were examined. The histological examinations, collagen deposition, and quantitative measurements of IL-1β, IL-6, and TNF in lung tissues and blood were investigated. Intraperitoneal, BLM administration (0.069 U/mL; 0.29 U/kg b.w.) for 33 days, caused IPF induction in Balb/c mice. Pulmonary combining therapy was administered with *L. minor* at dose 120 mg/mL (0.187 mg/kg b.w.). *L. minor* histologically ameliorated BLM induced IPF in lung tissues. *L. minor* significantly modulated (*p* < 0.05) BLM-alterations induced in lung hydroxyproline, carbonylated proteins, 5-MSL-protein oxidation. Oxidative stress decreased levels in antioxidant enzymatic and non-enzymatic systems in the lung were significantly regulated (*p* < 0.05) by *L. minor*. L. minor decreased the IL-1β, IL-6, and TNF-α expression in lung tissues and plasma. The *L. minor* improves the preventive effect/defense response in specific pulmonary protein oxidation, lipid peroxidation, ROS identifications, and cytokine modulation by BLM-induced chronic inflammations, and could be a good antioxidant, anti-inflammatory, and anti-fibrotic alternative or IPF prevention involved in their pathogenesis.

## 1. Introduction

Bleomycin (BLM) is a cytostatic glycopeptide antibiotic of the species Streptomyces verticillus. It has been clinically proven that BLM is used mainly as a chemotherapeutic due to lack of myelosuppression and immunosuppression [1]. The glycopeptide antibiotic is administered in malignancies, testicular cancer, Hodgkin’s lymphoma, but subsequently induces dose-dependent interstitial lung toxicity and lung tissue inflammation, which limits its clinical use [1,2]. BLM exerts its cytotoxic effect in vivo by cleaving the DNA structure in a biochemical process dependent on the presence of two cofactors—the molecular oxygen presence and the presence of metallic Fe (II) ion. Activated BLM conjugates both DNA and Fe (II). Oxidative degradation by molecular oxygen as a next step, converts Fe (II) to Fe (III) and generates DNA-cleaving reactive oxygen and nitrogen species (ROS/RNS, O_2_^•−^, H_2_O_2_, ^•^OH, ^•^NO), breaks the DNA chain and leads to cell death [1,3,4,5]. BLM-induced ROS and RNS decreased antioxidant status and dramatically increase fibroproliferation and extracellular matrix deposition. This favors the inflammatory mediators’ expression such as nuclear factor-activated nuclear cell enhancer (NF-κB), tumor necrosis factor (TNF), interleukin (IL)1, 6, 18, 22, 17a, inducible nitrogen oxide synthase (iNOS) [6,7], which completely destroy the lung architecture and induce fibrosis (PF). Investigations in lung-injury models support the IL-1, IL-6, TNF, and ROS as mediators of deregulated inflammation, implicated in the respiratory disorders pathogenesis [6,7,8,9]. Several studies have reported that ROS is directly involved in BLM-induced lung damage by inactivating endogenous and exogenous oxygen-sensitive enzymes, provoking genetic progression of interstitial fibrotic cells [8,9].

Protein carbonylation to aldo and keto aggregates leads to abrupt cellular accumulation and causes oxidative modifications and pulmonary cell dysfunction. Protein carbonylation is a major end product in various oxidative processes in the cell, and this makes it a suitable marker for studying the oxidative stress levels [10]. Cellular and tissues oxidative stress (OS) is caused by an imbalance between antioxidant/pro-oxidant processes, by ROS/RNS accumulation and by biological system disability to detoxify these reactive products [1,3,4,5,8]. Fernandez and Eickelberg [11] comment that fibrotic injury and OS increase myofibroblast synthesis and this leads to deposition of extracellular matrix proteins (ECM), including type I collagen and fibronectin in the lungs. Saito et al. [12] reported TGF-β-induced ROS stimulation and activation of mitogen-activated protein kinase signaling activation in pulmonary disorders. In addition, Hem oxygenase-1 (HO-1), as a stress-inducing protein, plays a protective role against oxidative upregulation in inhibiting fibrogenesis and improving lung fibrotic tissue [7,13]. During the early phase of lung damage, Steffen et al. [14] reported increased fibrotic albumin and cytokines (IL-1β; IL-6; IL-10) levels in bronchoalveolar fluid after BLM challenge.

Mast cells (MCs) derived from hematopoietic progenitors mature locally in lung tissue, and along with dendritic cells and macrophages, are among the first immune cells to be exposed to pro-inflammatory and toxic agents [15]. It is well-known that lung MCs synthesize mediators involved in the contraction of airway and lung smooth muscle cells, such as serotonin and ghrelin [16]. Inhibitory non-adrenergic non-cholinergic airway smooth muscle response is mediated by nitric oxide (NO) [17], and mast cells are a secondary source of NO-dependent relaxation of smooth muscle cells [18,19]. MCs accumulation in the lungs of patients with various forms of pulmonary fibrosis has been reported in a number of studies [20,21,22]. Histamine and renin of MCs cause locally formed ANG II [23] which is released near fibroblasts and leads to fibrogenesis. Veerappan et al. [23] describing the MCs involvement and fibroblasts in IPF found that interrupting this cycle by blocking MCs degranulation or blocking ANG II effectors and histamine receptors is crucial to prevent fibrosis. Earlier studies have documented BLM as an aggressive fibrous administrator leading to induction of membrane instability, increased lipid peroxidation, protein and cytokine expression, and pneumonia activation, in experimental animal models [24,25,26]. In addition, chronic BLM damage is a well-established experimental model resembling human IPF as it is characterized by increased OS [25,26], inflammatory cell infiltration, increased collagen content, and decreased lung sensitivity.

In animal models, BLM administration may be applied in a variety ways, including intraperitoneally, subcutaneously, intravenously, etc. [27,28]. Therefore, natural agents and drugs that inhibit fibroblast proliferation and collagen synthesis may be effective in clinical IPF treatment. Currently, the potential efficacy of new drugs in the preclinical IPF models is being investigated using a prophylactic rather than a therapeutic dosing regimen [29]. Usually, with great popularity in patients with proven IPF, histological evaluation and pulmonary collagen content are endpoints and no attention is paid to pulmonary changes in antioxidant/prooxidant balance [7,29].

Herbal agents (Quercetin, Curcumin, Resveratrol, Berberine, Withaferin A, Trigoneoside Ib, Curcuma longa, Tinospora cordifolia extracts and etc.) have been documented to be the new potentially effective oxidative inhibitors and antioxidant fibrous remodeling agents, which improve pulmonary fibrosis [6,7,25,26,30]. *Lemna minor* L. *(L. minor, Duckweed)*, a fast-growing freshwater plant used in traditional medicine as an antiscorbutic, depurative diuretic, natural agent effective for colds [31]. Structurally, *L. minor* extract contains 32 biologically active ingredients such as: phytosterols (>52.8 mg/kg), hydrocarbons (>23.1 mg/kg), aldehydes and ketones (>20.2 mg/kg), proteins (>21.80%), lipids (>11.1 mg/kg), etc., [32,33], determining the potential antioxidant nature [31]. Recently, it has been emphasized that *L. minor* is an environmental inhibitor that registers high toxicological and pharmaceutical potential [34]. The unique *L. minor* combined exposure to contaminants such as heavy metals, metal salts, paints, alkylbenzene sulfonate and synthetic drugs registered a decrease in the inclusion of 14C proteins, DNA, RNA remodeling, phospholipid damage, increased lipid peroxidation [35].

Plant protection systems are equipped with both enzymatic and non-enzymatic mechanisms to deal with ROS/RNS overproduction and tolerance to toxic stress. In addition, plants activate signal-regulatory molecules (proline) that stimulate many physiological or molecular responses needed to deal with cytostatic, metal, or salt-accumulated toxicity [36,37]. In a study of the anticancer drugs absorption in the aquatic environment, *L. minor* inhibited BLM concentrations up to >3 mg/L, and in binary mixtures up to 0.045 mg/L (33%) [5]. The *L. minor* adaptive mechanism to NH_4_^+^-induced oxidative stress is due in part to an additional antioxidant response that directly ROS scavenges and decompensates endogenous antioxidants, despite the reduced accumulation of soluble proteins and biomass in the plant [38]. The phytoremediation ability and stable *L. minor* antioxidant modification support normal electron flow in the respiratory electron transport chain, restore the mitochondrial electron transport chain (ETC) and reduce H_2_O_2_ overproduction after ciprofloxacin-induced oxidation [34]. In addition, Al-Snai [31] characterizes *L. minor* as an anti-inflammatory antioxidant capable of neutralizing ROS after acute administration in acute and chronic airway inflammation and autoimmune disorders. Most interestingly, rhamnogalacturonan-I (RG-I) domains containing pectin polysaccharides (PPs) side chains derived from *L. minor* have anti-inflammatory effects in acute colorectal mice, induced by 5% acetic acid administration [39,40]. Markov et al. [39] specify that PPs domains (homogalacturonan (HG), rhamnogalacturonan-II (RG-II), xyloglactauronan (XGA)) isolated from *L. minor*, as non-toxic natural anti-inflammatory products, have a significant anti-inflammatory function and reduce the colorectal lesion area. Transgenic plants such as *L. minor, S. tuberosum* L. and etc. have been required to activate the immune (Th1) response and to increase lymphoid organs protection [41,42].

In this study, we investigated for the first time whether *L. minor* exhibited protective therapeutic effects on bleomycin-induced pulmonary fibrosis in mouse models. Moreover, we examined the mechanism of *L. minor* extract action underlying the preventive effect against pulmonary fibrosis. Here, we hypothesize that *L. minor* prevents bleomycin-induced lung disorders and fibrosis in mice model by regulating levels of protein carbonylation and protein peroxidation, inhibiting the production of proinflammatory, pro-fibrous cytokines, and reducing oxidative disorders.

## 2. Materials and Methods

### 2.1. Plant Extract

*L. minor* was collected from natural water basins in Southern Bulgaria for 4 weeks. Initially, the green mass was washed twice with distilled water and air-dried at 22–25 °C for 24 h. The dried *L. minor* was ground and homogenized with an electric grinder (FP3121 Moulinex) to a fine powder. The powder mixture was dissolved in 2 L of distilled water and macerated with constant stirring for 48 h. Lyophilized aqueous *L. minor* was stored in the dark at 40 °C and 30% humidity. The protein content of 23.97% was quantified relative to the conversion of nitrogen content, using an automatic Kjeldahl system (Kjeltec 8200, Foss NIR-Systems, Delhi, India), registering a stable nutritional value of the Lemna species against the standard.

### 2.2. Chemicals

The Bleomycin Sulfate (C55H84N17O21S3, EP 9041-93-4/dose 0.35 U/kg), Dimethyl sulfoxide (DMSO), *N*-tert-butyl-alpha-phenylnitrone (PBN), xylene, paraffin, hydrochloric acid, thiobarbituric acid; phosphate-buffered saline (PBS, pH = 5.5; pH = 3.5; pH = 7.4), 5,5′-dithiobism (2-nitrobenzoic acid), 3-maleimido-2,2,5,5-tetramethyl-1-pyrrolidinyloxy (5-MSL) Nembutal, and commercial ELISA kits (Catalog No-CS0260, 2–80C) were purchased from Sigma Chemical Co., St. Louis, MO, USA.

### 2.3. Animals and Ethical Approval

Male Balb/c mice (*n* = 30) weighing 33–35.5 g aged 8–9 weeks were purchased from the Institute of Neurobiology, Experimental Breeding Base for Experimental Animals, Slivnitsa, Bulgaria. Male mice were chosen for the experiments because IPF is more easily induced in male and finds a BLM response regardless of age [43,44]. Conditions for keeping mice throughout the experimental protocol were: four animals per polycarbonate cage, temperature 21 ± 2 °C, relative humidity 50%, dark/light cycle 12:12 h, food: standard food for pellets, filtered water (pH = 5.5; ad libitum). The time from, approved by the Committee on Animal Ethics Animal procedures, the time of day for procedures were from 8 am to 6 pm, in accordance with Directive 2010/63/EU on the protection of animals used for experimental and other scientific work (172/6000-0333 19.05.2017).

### 2.4. Induction of Pulmonary Fibrosis and Therapeutic Protection

BLM hydrochloride (BLM) (0.069 U/mL; 0.37 U/kg body weight in 300 μL cold PBS, pH = 7.4) was administered i.p. in the lower abdomen with needle number 1 with steady breathing, twice per week for up to 33 experimental days. BLM hydrochloride was used to induce chronic toxicity and PF in mice [45] randomly divided into four groups, namely: (1) control group (*n* = 6) treated with 300 μL cold PBS, pH = 7.4 and standard diet; (2) BLM group (*n* = 12) treated with BLM dissolved up to 300μL in cold PBS, pH = 7.4 and standard diet; on day 16 after BLM administration *n* = 6 mice were separated and lung tissue was subjected to histological analysis; lung tissue and plasma was subjected to cytokine assays; (3) *L. minor* group (*n* = 6) (i.p. administration was performed at a concentration of 120 mg/mL (0.187 mg/kg b. w.) for 33 days, every two days, early in the morning, from 1 to 33 days); (4) BLM + *L. minor* group (*n* = 12) (administration i.p. was performed at a concentration of 120 mg/mL (0.187 mg/kg b. w.) for 33 days, every two days, early in the morning, 2 h before BLM administration). Injections were repeated four times weekly: (1) throughout the course, (2) for the first 16 days.

### 2.5. Analyze Lung Function

Mice were euthanized on day 34 by i.p. anesthesia with Nembutal 50 mg/kg and the lungs were perfused by injecting phosphate buffered saline (PBS) through the left aorta. Lung tissue was removed and fixed in ice, washed in cold PBS/4 °C, homogenized at 4000 rpm at 4 °C for 10 min and 2000 μL of supernatant was required for biochemical, immunological, and oxidative stress analyzes.

### 2.6. Histological Analysis for Visualization of Metachromatic Mast Cells

For histological examination, the right lung of each (*n* = 6) animal was immediately removed perfused, immersed in fixative 10% aqueous formalin solution for 24 h. After dehydration in a graduated series of ethanolic concentrations, the blocks are clarified in xylene and embedded in paraffin. The prepared 5 µm thick tissue sections were mounted on gelatin-coated slides dewaxed twice in xylene and rehydrated by a series of decreasing ethanol concentrations. Histological evaluation was performed by an independent observer after staining the sections with 0.1% toluidine blue and McLivane buffer (pH = 3) [46].

### 2.7. Histochemical Methods for Detection of Elastic and Collagen Fibers

Histological characteristics documentation and the prevalence of lung parenchymal lesions associated with original tissue loss and collagen fiber accumulation was studied by the Sirius Red, or Elastica-Van Gieson method [47].

### 2.8. Pulmonary Hydroxyproline Analysis

Analysis of hydroxyproline levels in lung tissue (drying at 110 °C for 24 h; hydrolysis with 6N HCl, incubation at 110 °C) was used to quantify the level of fibrosis. For this purpose, pulmonary hydroxyproline was determined spectrophotometrically, by 550 nm absorption, by a method with slight modifications [9,48]. The results obtained are expressed as μg of hydroxyproline per gram of tissue used.

### 2.9. Protein Carbonyl Content (PCC), Glycation End Products (AGEs) and 3-Maleimido Proxyl (5-MSL) Protein Oxidation Analysis

Protein oxidative damage (PCC) was assessed by determining carbonyl groups based on reaction with dinitrophenylhydrazine (DNPH) to DNP hydrazone (2 h at 37 °C), as assessed by the OxiSelect Total Carbonyl Protein ELISA Kit (Cell Biolabs). Protein carbonyl content was determined based on oxidized/reduced BSA standards at 370 nm absorption, and carbonyl derivatives were expressed as nmol/mg. Monitoring of Advanced Glycation End Products (AGEs) levels was assessed similarly to PCC with the OxiSelect AGE Competitive ELISA Kit (Cell Biolabs). The AGE-protein content in unknown samples was determined by comparison with a predetermined AGE-BSA standard curve (nmol/mg).

The degree of protein/albumin damage in lung tissue was assessed by the in vivo EPR method using spin-conjugation with 3-maleimido proxyl (5-MSL). Lung tissue (10 mg) was added to 900 μL of 20 mM 5-MSL dissolved in DMSO and the homogenate was centrifuged at 1000 rpm for 15 min at 4 °C. The protein/albumin SH content was assessed by triplicate measurement of the recorded in vivo EPR spectra (at 3505 G, 6.42 MW power, 5 G modulated amplitude, 12 modulation, at 3 scans), in random units, by the method described earlier [49].

### 2.10. Lipid Peroxidation and Endogenous Antioxidant Enzyme Activity Analysis

Lipid peroxidation in lung tissue was assessed by the method of Plaser et al. [50], against equivalent concentrations of malondialdehyde (MDA nmol/mg protein; TERMO Sci., RS232C, USA). Overall ROS overproduction in lung tissue by in vivo EPR method (X-Band, Emxmicro Spectrometer, Bruker, Germany), to 900 mg of lung tissue was added 900 μL of 50 mM PBN dissolved in DMSO and centrifuged at 4000 rpm for 10 min at 4 °C. ROS products were evaluated by triple measurement of the recorded EPR spectra, in random units, by a method described previously [51]. Pulmonary catalase (CAT) activity was assessed by the Aebi method [52], at an absorbance of 240 nm. The activity of cellular pulmonary superoxide dismutase (SOD) was analyzed by Sun et al. [53], at 420 nm absorption. Glutathione peroxidase (GPx) levels in lung cells were assessed by Akerboom and Sies [54]. Decreased GSH is assessed by a continuous decrease in DTNB, expressed as nmol of GSH per milliliter of protein, at 412 nm absorption.

### 2.11. Measurement of Cytokine Assays (IL-1β, IL-6, TNF-α)

Mouse IL-1β, IL-6 and TNF-α concentration were determined in lung homogenate of right/left lobes and blood plasma (centrifugated for 10 min, 4000× *g*) by using the ELISA Kits (Bio-Science) according to the manufacturer’s protocols, for the period of 1–16 day and 16–33 day the experiment.

### 2.12. Statistical Analysis

Mast cell number was determined on a microscopic field × 200 with an area of 0.163 mm^2^ of sections of the right lung per each animal using a light research microscope (LEICA DM1000) equipped with a digital camera (LEICA DFC 290). Interalveolar septa thickness in all groups was estimated as well. Mast cell density (number/field of view) and interalveolar septa thickness data were processed by one-way ANOVA, followed by Tukey Kramer test (GraphPadPrism 6 for Windows; GraphPad Software, San Diego Inc., San Diego, CA, USA) for variation analysis. Values of *p* ˂ 0.05 were considered statistically significant. The data are given as mean ± standard deviation (SD).

The remaining statistical analyzes were performed using Excel Version 10.0 software, StaSoft, Inc., San Diego, CA, USA and presented as mean ± standard error (SE). Results of the EPR spectral processing were performed using Win-EPR and Sim-fonia Software as averages of three replicates. Statistical analysis was performed using a student *t*-test to determine differences. A value of *p* < 0.05 is considered statistically significant.

## 3. Results

### 3.1. Body Weight and Lung Histopathology

The body weight changes in BLM-induced lung changes/fibrosis and in other 3 groups were measured at 5th, 9th, 16th and 33rd days during the experimental 33 days periods (Table 1). The results showed that the body weight of BLM-treated mice was significantly reduced compared to controls at all tested period (*p* < 0.05, *t*-test) by as much as approximately 12% by day 16, and 23.5% by day 33, compared to the controls. The *L. minor* administration (120 mg/kg) significantly reduced BLM-induced weight loss and animals are significantly better than controls (*p* < 0.005, *t*-test)

**Figure 1:**
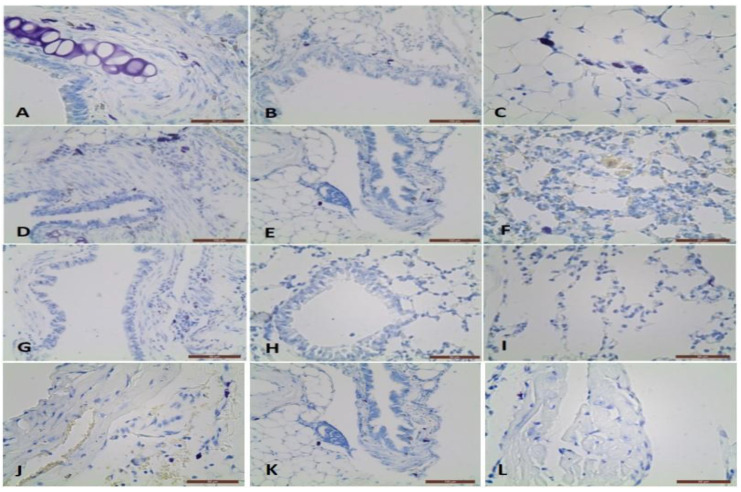
Metachromatical mast cells in the propria, muscle layer in the large bronchus adventitia, small bronchus adventitia, and interalveolar septa near the capilaries: (**A**–**C**) in BLM treated BALB/c mice; (**D**–**F**) in BLM + *L. minor* treated BALB/c mice; (**G**–**I**) in control BALB/c mice; Metachromatic mast cells in the artery adventitia in BLM treated group (**J**), in BLM + *L. minor* group (**K**) and controls (**L**), respectively.

Toluidine-blue stained lung sections were examined to determine whether BLM-induced lung damage was reduced after antioxidant treatment. In order to study lung changes we identify metachromatic MCs in the airways wall and blood vessels (arteries, veins, venules, arterioles, and capillaries) (Table 2), (Figure 1). We employed a BLM model to investigate the *L. minor* effect on MCs. The MCs number was the highest in the large cartilaginous bronchi wall, followed by small bronchi, blood vessel adventitia, and near the capillaries, in all tested groups.

The MCs in the large bronchi’s wall was the highest in BLM group (*p* ˂ 0.05), followed by BLM + *L. minor* group and the controls (Table 1, A4, *p* ˂ 0.0001/B2, *p* ˂ 0.01). MCs were localized predominantly in the adventitial and the airways muscle layers. In BLM administrated animals 30% of MCs were found in the large bronchi propria, while in other two groups MCs were observed mainly in the muscle layer and adventitia. In the small bronchi wall, MCs number was similar in both groups BLM and BLM + *L. minor*, while in the control were not identified. MCs were not observed in the terminal bronchioli in four treated groups. In the blood vessel’s wall, MCs were identified mainly in the adventitial layer is presented on Figure 1J–L. The most MCs were detected in the arteries adventitia and veins as well as near the lung capillaries in the BLM group. The combined BLM + *L. minor* use may reduce MCs, inflammatory infiltration and alveolar destruction and may prevent histopathological changes in chronic BLM induction. It is important to note that in the interalveolar septa, MCs number was highest in the BLM group (Table 1, A3 *p* ˂ 0.001). However, in BLM + *L. minor* group and controls, single cells -were observed. The MCs number per area of lung was increased with BLM-administration and was inhibited by *L. minor*. These data correlated with the thickness of the interalveolar septa which was largest in the BLM group, but it decreased in the BLM + L. minor group and was lowest in the controls (Table 1) (Figure 1C,F,I). No statistical significance was detected between the BLM + *L. minor* group and controls.

**Figure 2:**
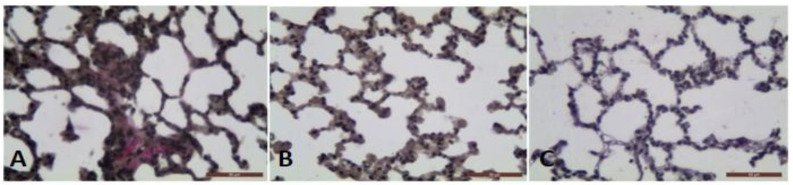
The photomicrographs of lung sections (middle pulmonary field) obtained from male BALB/c mice. Groups: (**A**) mice with BLM induced fibrosis and intensive red staining of collagen deposition, (**B**) mice with BLM induced fibrosis treated with *L. minor* and non-collagen deposition; (**C**) controls, non-collagen deposition. Tissues stained by Van Gieson, scale bar 50 μm; *p* < 0.001 significant difference compared (**A**) with (**B**,**C**).

Van Gieson histochemical method allowed the identification of the intensive collagen deposition in the interalveolar septa of BLM treated animals with fibrosis (Figure 2A). The collagen deposition decreased significantly in the BLM + *L. minor* group ((*p* = 0.001, *t*-test) Figure 2B). There was no significant staining in the lung parenchyma of controls (Figure 2C).

### 3.2. Pulmonary Hydroxyproline Analysis

**Figure 3:**
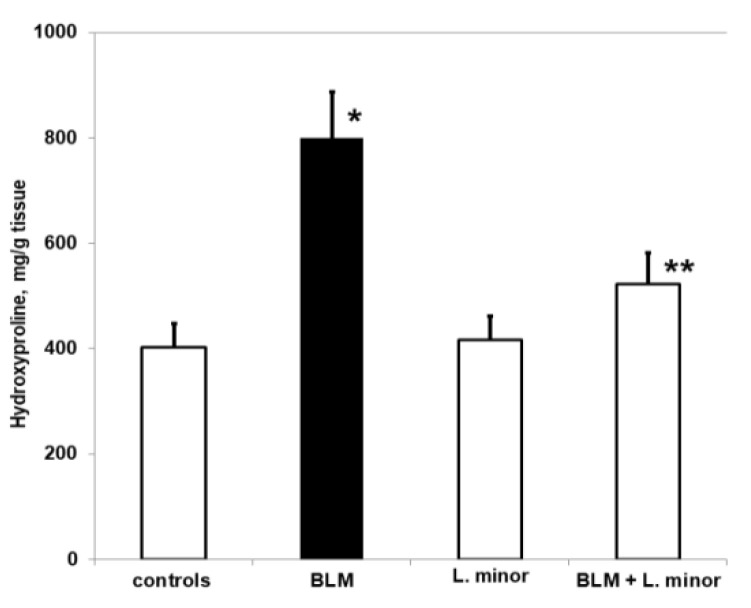
The *L. minor* effects on BLM-induced oxidative changes in lung hydroxyproline content. Values are presented as mean ± SEM and results are expressed in μg/gr tissue hydroxyproline. The significant difference used in relation to controls (*) *p* < 0.03 vs. controls, (**) *p* < 0.02 vs. BLM.

Pulmonary-induced toxicity and IPF were assessed by measuring the hydroxyproline content of lung tissue, i.e., as a collagen accumulation index. The hydroxyproline content of the four study groups is shown in Figure 3.

Chronic BLM administration resulted in a significant increase in hydroxyproline levels compared to controls (*p* < 0.005, *t*-test). The BLM-induced increase in pulmonary hydroxyproline was statistically significantly reduced after treatment with *L. minor* (*p* < 0.0002, *t*-test). However, animals treated with *L. minor* for 33 days showed lower levels of pulmonary hydroxyproline compared to controls (*p* < 0.05, *t*-test). There was a significant reduction in collagen accumulation in lung tissue compared to *L. minor*, and BLM + *L. minor* treated mice, respectively *p* < 0.002, *p* < 0.005 *t*-test.

### 3.3. Determination of Oxidative Protein Remodeling in Lung Tissue

**Figure 4:**
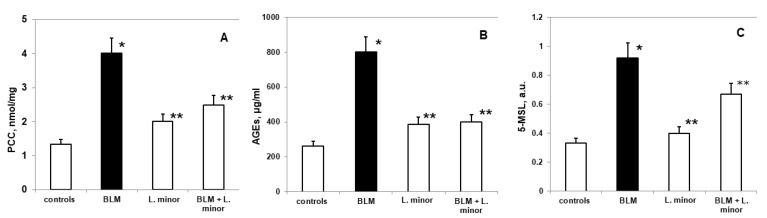
The *L. minor* effects on BLM-induced oxidative changes in protein carbonyl content (PCC, (**A**)), advanced glycation end products (AGEs, (**B**)) and 5-MSL protein oxidation (**C**). Statistically significance was higher than controls, *p* < 0.05, *t*-test. The results are presented as mean ± S.E. *p* < 0.05; (*) vs. to controls; (**) vs. BLM.

The lungs are highly susceptible to oxidative remodeling, including BLM-induced chronic toxicity and IPF induction. Figure 4A shows the changes in PCC in lung tissue of BALB/c mice treated with *L. minor* at a dose that we found to be sufficient to prevent pulmonary fibrotic remodeling.

Statistically significant increased oxidative changes in pulmonary PCC was reported after i.p. application of BLM compared to controls (mean 4.02 ± 0.05 nmol/mg vs. mean 1.33 ± 0.04 nmol/mg, *p* < 0.02, *t*-test). A statistically insignificant increase in PCC was observed in the BLM + *L. minor* treatment group, compared to controls (mean 2.49 ± 0.02 nmol/mg vs. mean 1.33 ± 0.04 nmol/mg, *p* < 0.02, *t*-test). *L. minor* administration (120 mg/kg) significantly inhibited BLM-induced PCC expression compared to the BLM-controlled group (mean 2.49 ± 0.02 nmol/mg vs. 4.02 ± 0.05 nmol/mg, *p* < 0.02, *t*-test). There was significant carbonylation of lung protein in controls and BLM-treated mice, *p* < 0.003, *t*-test.

A statistically insignificant increase in AGEs (Figure 4B) was observed in BLM-treated mice compared to controls (802.3 ± 11.69 μg/mL vs. 261.1 ± 9.05μg/mL, *p* < 0.05, *t*-test). There was a statistically insignificant increase compared to controls: *L. minor* (120 mg/kg) (385.4 ± 10.6μg/mL, vs. 261.1 ± 9.05μg/mL, *p* < 0.001, *t*-test) and BLM + *L. minor* (399 ± 11.1μg/mL, vs. 261.1 ± 9.05μg/mL, *p* = 0.01, *t*-test). Both groups pretreated with *L. minor* and BLM + *L. minor* combination showed a statistically significant reduction in advanced glycation end products (AGEs) to BLM group (*p* < 0.05, *t*-test; *p* < 0.004, *t*-test).

Nitroxides are predominantly distributed in the lung region [49] and the reading of available oxidative proteins remodeling is assessed by in vivo EPR [49], using spin-conjugation with 5-MSL, as performed in the present study (Figure 4C). Compared to controls, the expression of pulmonary 5-MSL-conjugated proteins was significantly increased (0.332 ± 0.04 a.u. vs. 0.924 ± 0.1 a.u., *p* < 0.005, *t*-test) after controlled administration of BLM. *L. minor* treatment (120 mg/kg) significantly reduced (*p* < 0.05) BLM-induced pulmonary protein up-regulation in lungs compared to BLM-controlled mice (0.62 ± 0.04 a.u., versus 0.924 ± 0.1 a.u., *p* < 0.05, *t*-test). However, *L. minor* administration (120 mg/kg) resulted in pulmonary expression inhibition of 5-MSL-conjugated proteins, the value being comparable to controls (0.332 ± 0.04 a.u., versus 0.4 ± 0.06 a.u., *p* < 0.05, *t*-test). Conjugation of lung/fibrotic proteins differed significantly in controls and BLM-treated BALB/c mice, respectively, *p* < 0.005, *t*-test.

### 3.4. Parameters of Oxidative Damage and Antioxidant Enzyme Activities in Lung Tissue

**Figure 5:**
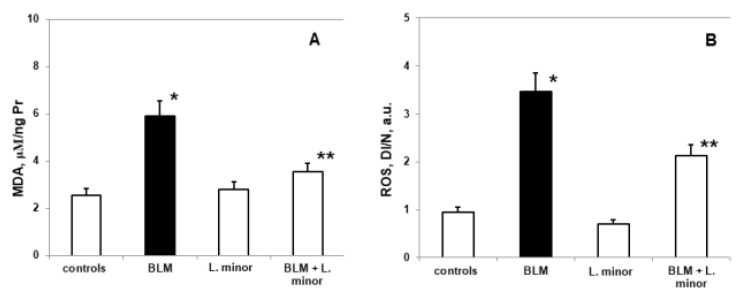
The *L. minor* effects on BLM-induced oxidative changes in malondialdehyde concentration (MDA, (**A**)) ROS production (**B**). Statistically significance was higher than controls, *p* < 0.05, *t*-test. The results are presented as mean ± S.E. *p* < 0.05; (*) vs. to controls; (**) vs. BLM.

We evaluated lipid peroxidation and ROS production (Figure 5) as indices of BLM-induced oxidative damage in the lungs and the oxidative stress modulate response of the antioxidant defense system.

**Figure 6:**
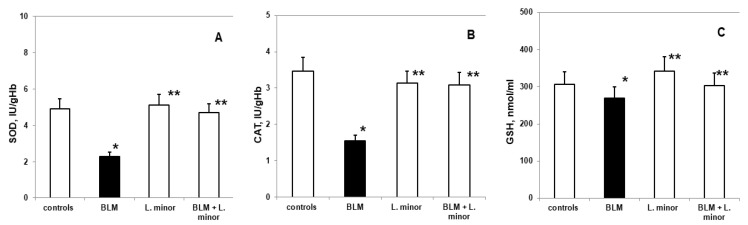
The *L. minor* effects on BLM-induced oxidative changes in catalase (CAT, (**A**)), superoxide dismutase (SOD, (**B**)) and glutathione (GSH, (**C**)). Statistically significance was higher than controls, *p* < 0.05, *t*-test. The results are presented as mean ± S.E. *p* < 0.05; (*) vs. to controls; (**) vs. BLM.

Compared to controls, SOD levels (2.285 ± 0.35 IU/gHb vs. 4.92 ± 0.86 IU/gHb, *p* < 0.05), CAT (1.59 ± 0.13 IU/gHb vs. 3.48 ± 0.05 IU/gHb, *p* < 0.003) and GSH (307 ± 22.86 nmol/mL vs. 269.8 ± 11.35 nmol/mL, *p* < 0.05) decreased significantly, while MDA levels (5.92 ± 0.73 IU/gHb vs. 2.56 ± 0.49 IU/gHb, *p* < 0.05) and ROS products (3.77 ± 0.46 a.u., vs. 0.95 ± 0.25 a.u., *p* < 0.002) increased statistically significantly in the lungs of i.p. BLM-controlled animals, after IPF induction. *L. minor* administration showed significant (*p* < 0.05) inhibition of BLM-induced oxidative damage and antioxidant defense system recovery (Figure 6) in lung tissue compared to BLM controlled mice: SOD (4.68 ± 0.72 IU/gHb vs. 2.285 ± 0.35 IU/gHb, *p* < 0.05); CAT (3.09 ± 0.8 IU/gHb vs. 1.59 ± 0.13 IU/gHb, *p* < 0.003); GSH (303.5 ± 15.1 nmol/mL vs. 269.8 ± 11.35 nmol/mL, *p* < 0.05); MDA (3.44 ± 0.99 IU/gHb vs. 5.92 ± 0.73 IU/gHb, *p* < 0.05); ROS products (2.03 ± 0.22 a.u. vs. 3.77 ± 0.46 a.u., *p* < 0.002). *L. minor* self-administration (120 mg/mL) statistically non-significantly increased SOD, GSH and MDA levels, while decreasing CAT and ROS levels in the lungs compared to controls.

### 3.5. Pulmonary and Plasmatic IL-1β, IL-6, and TNF-α Concentration in BLM-Damaged Lungs and Protective L. minor Combination

**Figure 7:**
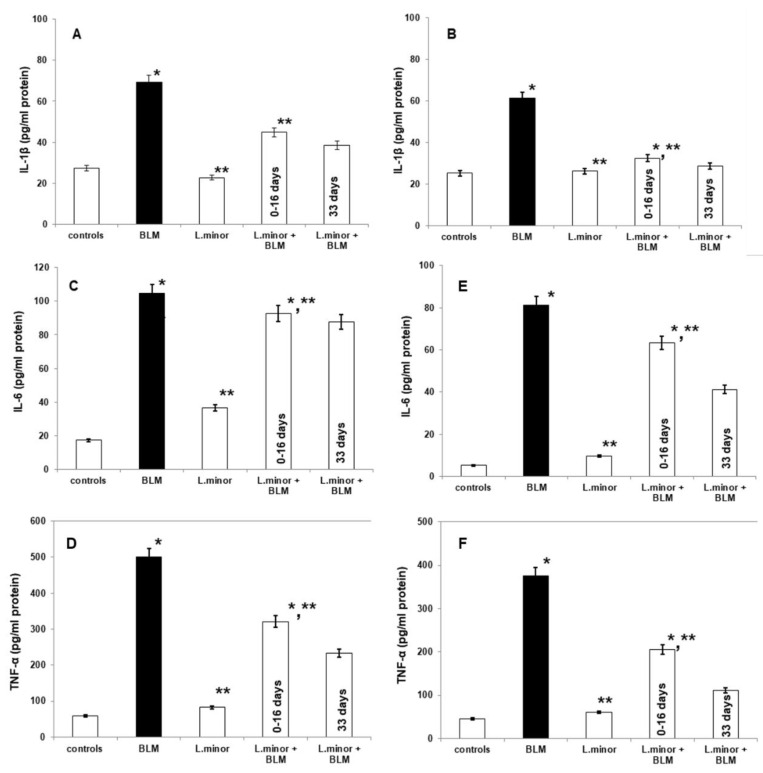
The *L. minor* effects on BLM-induced pulmonary fibrosis and the expressions of IL-1β, IL-6, and TNF-α in mouse lung homogenates (IL-1β, IL-6 and TNF-α, (**A**,**C**,**D**), respectively) and plasma (IL-1β, IL-6, and TNF-α, (**B**,**E**,**F**), respectively). The *L. minor* effect was investigated in two ways: (1) throughout the 33 day course (2) for the first 16 days. The results are presented as mean ± S.E. *p* < 0.05; (*) vs. to controls; (**) vs. BLM.

BLM administration increase lung concentrations of IL-1β (69.16 ± 2.69 vs. 27.36 ± 1.37, *p* < 0.005, *t*-test), IL-6 (104.7 ± 13.45 vs. 17.466, *p* < 0.02, *t*-test), and TNF-α (500.09 ± 42.19 vs. 58.84 ± 4.51, *p* < 0.05, *t*-test) observed in BLM-induced fibrosis group, compared to controls (Figure 7A,C,E). Notably, *L. minor* lung protection markedly decreased in the IL-1β levels (by 38.82 ± 5.41, *p* < 0.0003, *t*-test) and TNF-α levels (by 232.87 ± 35.09, *p* < 0.0062, *t*-test), compared to BLM-induced fibrosis group, on the course 33 days, respectively. BLM + *L. minor* reduced IL-6 lung concentration (by 92.63 ± 4.11 day 0–16) 87.7 ± 5.23, on the 33 day course with statistical significance (*p* < 0.005, *t*-test). Moreover, monotherapy by *L. minor* protect the connective tissue deposition in the lungs which is evident from the statistically insignificantly decreased or commensurate to the control levels of the lung fibrosis-related IL-1β, IL-6, and TNF-α concentration, respectively. To confirm the *L. minor* mediated protective effect the inflammatory cytokines were investigated in blood plasma (Figure 7B,D,F). Plasmatic IL-1β (61.23 ± 3.15 vs. 25.17 ± 2.44, *p* < 0.003, *t*-test), IL-6 (81.14 ± 11.37 vs. 5.22 ± 0.932 *p* < 0.0053, *t*-test), and TNF-α (376.38 ± 26.619 vs. 45.54 ± 5.68, *p* < 0.002, *t*-test) expressions were significantly up-regulated in BLM-controlled mice, in compare to controls.

Notably, *L. minor* protection (120 mg/mL) significantly down-regulated plasmatic IL-1β, IL-6 and TNF-α expression at the first 16 days and at last 16 days (Figure 7), compared to BLM-administration. BLM-induced plasmatic alterations in IL-1β (27.03 ± 1.56 vs. 61.23 ± 3.15, *p* < 0.0036, *t*-test), IL-6 (41.19 ± 2.26 vs. 81.14 ± 11.37, *p* = 0.0042, *t*-test), and TNF-α (111.07 ± 6.14 vs. 376.38 ±26.619, *p* < 0.002, *t*-test) expressions were two-fold significantly attenuated *L. minor* after pretreatment, at last 16 days (*p* < 0.05). Moreover, the *L. minor* attenuation of plasmatic inflammations and BLM-fibrotic alteration in IL-1β expressions were almost comparable to controls (25.17 ± 2.44 vs. 27.03 ± 1.56, *p* = 0.001, *t*-test).

### 3.6. Positive Correlations between Parameters

A positive correlation was observed for PCC compared to AGEs (r = 0.95, *p* < 0.02); PCC versus 5-MSL captured protein (r = 0.91, *p* = 0.001); MDA vs. PCC (r = 0.93, *p* = 0.01); MDA versus AGEs showed (r = 0.92, *p* < 0.05); MDA vs. ROS showed (r = 0.91, *p* < 0.002); GSH versus ROS showed (r = 0.91, *p* = 0.01); GSH versus 5-MSL captured protein (r = 0.93, *p* = 0.01). IL-1β versus TNF-α showed (r = 0.81, *p* < 0.004); PCC compared to IL-6 (r = 0.85, *p* < 0.05).

## 4. Discussion

The antigen-specific flavonoids, amino acids, and proteins presence as structural *L. minor* components and high antioxidant activity make the duckweed suitable to stimulate and integrate the immune response and as a protector in pharmaceutical intakes [55,56]. The duckweed/*Lemna species* were used for acute nephritis and inflammation treatment in Asian countries [57]. Cardoso et al. [58] determine low or missing cellular necrosis on human immune cells after 48 h *L. minor* treatment.

Biologically active proteins and amino acids present in *L. minor* determine immunomodulatory potential against a specific protein ovalbumin antigen, which emphasizes antimicrobial activity against bacterial and fungal strains [59]. Ko et al. [60] commented the transgenic *L. minor* support for the protective antigen expression of the epidemic diarrhea virus (PEDV) in pigs. *L. minor* (containing 1.96% total soluble proteins) has been used successfully to express the M2e gene of influenza virus (H5N1) in birds [61]. In Cox et al., research [62], *L. minor* was used to express human monoclonal antibody (mAb) antibody optimized by RNA interference. The authors comment on the therapeutic *L. minor* protein concentration, without zoonotic pathogens, glycosylation homogeneity and cell-mediated cytotoxicity. Yang et al. and Dickey et al. [56,63] summarize that transformed duckweed/*Lemna* species expresses bioactive protein hormone (interferon-α2 reaches > 30% protein level), growth factor, insulin and human growth hormone (human auxin > 609 mg/L).

In addition, Whitlow et al. and Yadav et al. [41,42] highlight the *L. minor*, *S. tuberosum* L. and etc., on antigens expressed vaccination therapy, required to activate the immune (Th1) response and to increase lymphoid organs protection against lungs *Mycobacterium tuberculosis* in human.

Based on these facts, we aimed to establish for the first time the possible inhibitory and protective effect of freshwater *L. minor* (120 mg/mL) extract, to reduce lung inflammation, idiopathic lesions and to modulate lung oxidized proteins and oxidative disorders in BALB/c mice exposed to progressive BLM-induced IPF. In adition, we investigated the *L. minor* potential regulative mechanism of inflammatory cytokines expression in lungs and plasma at the 16 days, 33 days BLM-administration.

The BLM induced lung damages progression and pulmonary fibrosis is characterized by inflammation, the excessive extracellular matrix deposition [64]. Shieh et al. [59] point out that lung damage in mice is caused by the pathogenic role of the early inflammatory response, increased uric acid deposition and selective modulation of key cytokine inflammatory pathways.

Initial results indicated a sharp decrease in body weight, appetite, dyspnea, increased collagen accumulation (Figure 3) and histopathological changes in the lungs after BLM administration associated with typical clinical features of IPF (Figure 1). Studies by various teams confirm our results, which report that BLM administration increases pulmonary inflammatory influx promotes collagen deposition in lung cells [1], and alters the integrity and efficiency of the alveolar capillary membrane [7,27,65]. Accordingly, the antioxidants, improves body weight, appetite, breathing difficulty, and reduces the inflammatory response after ROS neutralization generated in BLM-induced IPF in animals [7,66,67]. Moreover, the presented results show that 33 days course *L. minor* combined therapy and statistically significantly prevent the fibrotic process provoke a decrease in the chain inflammatory response, reduces the extracellular matrix and fibroblast proliferation, and therefore improves the general body condition. In addition, *L. minor* daily inclusion significantly reduced the metachromatic mast cells density, especially in the interalveolar septa and large bronchial wall, with BLM + *L. minor* values close to controls [33]. Intraperitoneal administration of lemnan, the major apiogalacturonanic pectin in *L. minor*, has been shown to improve the serum titer of specific IgG antibodies, which identifies *L. minor* extract as a possible potent modern mucosal adjuvant that restores protein antigens [68]. Therefore, the *L. minor* addition has a beneficial effect on BLM-induced inflammation and fibrotic processes, possibly participating in the Th1/Th2 immune response modulation and the subsequent decrease in the MCs and eosinophils set. Our results are supported by reports from a number of authors on the crucial role of mast cells and the Th2 immune response in IPF progression [16,17,18,21,22,23,69]. The accumulation of collagen together with increased MCs number in the interalveolar septa in BLM treated group detected in the current study correlates with the findings of several authors suggested that the MCs mediators tryptase, chymase and histamine induce lung fibroblast proliferation and collagen production [23,70,71,72,73] recently demonstrated that stretch-induced degranulating MCs activate the pro-fibrotic cytokine TGF-β1.

Veerappan et al. [23] demonstrated that mice genetically deficient in MCs are protected from BLM-induced fibrosis. The in vitro studies in lung fibroblasts show that histamine and ANG II promote fibroblast proliferation, TGF-b1 secretion, and collagen synthesis via activation of histamine H1 receptors and the ANG II AT1Rs, respectively. The increase of the interalveolar septa thickness together with the elevated MCs number in fibrotic area of BLM-treated animals observed in the current study correlates with the findings of other authors established that pulmonary fibrosis is characterized by an infiltration of the parenchyma by inflammatory cells, including mast cells [23,74,75,76] followed by an increase in extracellular matrix production due to proliferation and fibroblasts activation [77]. Overed-Sayer et al. [78] reported that nintedanib inhibits MCs survival both in vitro and in vivo via ckit (also known as CD117 or SCF receptor); however, the efficacy of nintedanib in IPF likely resides in the drug’s ability to modulate multiple fibrotic pathways. According to Wolin et al. [79] for nintedanib, these positive effects include effects on VEGF receptors, fibroblasts and platelet GF, tyrosine kinases (Src, Flt3, Lyn and Lck) [80] and suggest that effects on MCs function may be mediated by additional tyrosine kinases [81]. Understanding the L. minor anti-fibrotic mechanisms of action by decreasing MCs density could also enable the discovery of better novel therapies that more specifically target pro-fibrotic mechanisms and minimize the BLM-therapy side effects.

BLM application accumulates ROS production, unwanted protein oxidation and OS induction. It can be assumed that BLM administered cellular inflammation is a consequence of activated inflammatory cells over-accumulation (alveolar macrophages and neutrophils) in the lower respiratory tract [8,9], which produce the cellular ROS generation by an iron-dependent mechanism [8,9]. The presence of an iron-dependent mechanism during the inflammatory process caused by BLM-accumulation increases hydrogen peroxide (H_2_O_2_) and hydroxyl (^•^OH) and lipid radicals, respectively [8,9]. In addition, the *L. minor* extract (120 mg/mL; 23.97 % protein content) used in our study possessed antioxidant activity and modulate redox imbalance [55]. Existing data identify *L. minor* as an adequate phytoremediator that regulates the activity of antioxidant systems (CAT, APX and substrate ascorbate), thus ensuring that H_2_O_2_ and ^•^OH concentrations are maintained below phytotoxic levels, i.e., without disrupting electron transport activity and cellular metabolism [31,34]. Therefore, *L. minor* can be used not only as a phytoremediating plant, but also as a protector that directly inhibits elevated OS, regulates protein oxidation and collagen deposition, reduces BLM induced lung lesions and can improve IPF response.

Carbonyl stress induces complete protein dysfunctions and pathologically damages lung tissues, contributing to the acceleration of inflammation and the IPF development [82,83]. Cameli et al. [67] found increased carbonylated oxidation of specific proteins or substrate accumulation of reactive dicarbonyls activated only in IPF patients. Vásquez-Garzón et al. [82] commented on irreversible protein oxidation, increased protein proliferation markers and inflammation, lipoperoxidation, and OS disorders induction in redox status of 35 days treated mice with 100 U/kg BLM. Endogenous lipid peroxidation strategically increases the PCCs formation in parallel with carbohydrate glycation processes (precursors of advanced glycation end products (AGEs)). The tissue proteins accumulation registers carbonyl stress during chronic induced disease [67,82,83], as in the IPF case. These our results are in complete agreement with previously reported studies [82,83], and demonstrated that in the BLM treated group there was a statistically significant twofold increase in PCC (Figure 4A), AGEs (Figure 4B) and delayed reduction in nitroxide protein distribution (Figure 4C), which leads to the IPF registration. It is important to note that the combination treatment with BLM + *L. minor* significantly reduces these parameters. Our study suggests that aqueous *L. minor* extract, due to the high active proteins and amino acids content in its structure stimulates antigen-specific immune response, which restores protein oxidation and determines inflammations and cell-mediated cytotoxicity [59,62]. Therefore, *L. minor* modulates BLM-induced inflammation by reducing carbonyl stress, reducing protein dysfunction and completely inhibiting the O_2_^•−^, ^•^OH radicals’ concentrations in the lung and fibrotic lesion area. This result is consistent with the fact that *L. minor*, as non-toxic natural anti-inflammatory product, have anti-inflammatory function, reduce the colorectal lesion area [39] and protect lungs against *Mycobacterium tuberculosis* in human [41,42]. In addition, it rapidly inhibits AGEs glycation and possibly inhibits the action of long-lived proteins and non-protein thiols [1]. At the same time, *L. minor* inactivates PCC oxidation and inhibits procoagulant activity in alveolar spaces and normalizes collagen turnover [1,84] through changes in antioxidant/prooxidant balance in pulmonary cells.

Fibrinogen degradation products (FDPs) are secondarily deposited around the perimeter of the cancerous tissue [85]. In addition, lung carcinoma tissues contain insoluble forms of human serum albumin. Based on these, Kieliszek, Lipinski, ref. [85] hypothesized that insoluble fibrinogen albumin complexes cover tumor cells and present them as to natural killer cells, as redox-active selenium or by the amphiphilic natural polyphenols. Extravascular accumulation of pulmonary fibrin in acute and chronic lung diseases induced in animals is probably caused by the ^•^OH disruption, leading to fibrinogen modification [85]. Takeshita et al. [49], summarizes that once 5-MSL captures membrane albumin/protein, it leaves the maleimide group to react with the SH regions until alkylation, i.e., the rotational nitroxyl movement captures the weakened -SH regions of the corresponding amino acid. Based on these findings, we hypothesize that the *L. minor* therapeutic effect (120 mg/mL) is due to a modulated cellular response from the high polyphenol content [59,82], leading to a membrane albumin/protein reduction, ^•^OH reduction and an amyloid protein aggregates neutralization.

In the BLM-induced IPF model, ROS generation leads to progressive lipid peroxidation, which affects the reductive carbonyl compounds metabolism, DNA damages, increases collagen synthesis, and directly affects the antioxidant defense system [86]. ROS deactivation is performed by endogenous and exogenous antioxidant systems (SOD, CAT and GSH and etc.). Antioxidant enzymes catalyze the reaction of O_2_^•−^ dismutation, and their inability to reduce with oxidative disorders leads to the development of many pathological conditions [9]. Intracellular ROS and OS infiltration after BLM-induced pulmonary fibrosis is performed after the various antioxidants addition [86]. In accordance with previous studies [7], our results also show that the BLM application leads to a statistically significant increase in the parameters of OS damage i.e., increased lipid peroxidation (MDA) and ROS production (Figure 5), along with increased protein carbonylation. The depletion of the antioxidant enzymes SOD, CAT and GSH activity (Figure 6) in lung tissue indirectly reflects the intracellular ROS generation and OS damages. Also, there was a statistically significant increase in the SOD, CAT and GSH activity (Figure 5) and a decrease in the lipid peroxidation levels and residual ROS (Figure 6) in the BLM + *L. minor* combination. The lipid peroxidation as tissue damaging mechanism provokes paracellular permeability increase in Caco-2 cell monolayers [68]. Statistically *L. minor* enhancement of MDA concentration and ROS production in pulmonar tissue suggested fixation of epithelial cellular barrier function. Our results are in agreement with reports of Popov et al. [68], show that lemnan, *L. minor* isolated apiogalacturonanic pectin, manifests increased mucosal adjuvanticity result from an intestinal epithelial lipid barrier alteration. Therefore, *L. minor* manages to reduce lung tissue damage and affect fibroblast stimulation by reducing O_2_^•−^ concentrations in the extracellular space and balancing the H_2_O_2_ content and POS products after lipid peroxidation. In addition to our results, Pagliuso et al. [55] hypothesizes that C-glycosylated (luteolin-8/6-C; apigenin-8/6-C and etc.) flavonoids predominant presence in the *duckweed/Lemna species* probably protect against induced OS disorders in endogenous/exogenous system were prevention action, responsible for heart diseases and cancer. The vitexin and apigenin in *duckweed* have been suggested as constituents for treating non-small lung cancer [55] and as anticancer adjuvants, and flavone *C*-glycosides from *L. japonica* exhibits cytotoxic activity against various human cancer cell lines (HepG-2, SW-620, A-549) [87]. In particular, *L. minor* flavonoids have immunosuppressive effects by reducing free hemoglobin content and antibody production in human whole blood [87]. Moreover, fruit and vegetable consumption rich in polyphenolic substances and iron chelating agents prevent cancer by ROS scavenging [85].

Earlier studies have documented the pro-inflammatory cytokines overexpression being associated with BLM induced lung fibrosis in animal models, including IL-1β, IL-6 and TNF-α [7,88]. Bale et al. [89] comment the pro-inflammatory cytokines ability to induce the fibroblast production and extracellular matrix synthesis. IL-6 mediates many inflammatory processes in the mice and humans lungs, and IL-6 blocking unregulated release has been implicated in the fibrosis pathogenesis [64,90]. IL-6 increased circulation regulate muscle mass by decreasing protein synthesis, high Atrogin-1 protein expression in the quadriceps muscle tissue and STAT3 signaling activation in BLM treated mice [64,91]. IL-1β is a potent pro-inflammatory mediator, produced after the most frequent activation of the nucleotide-binding oligomerization domain-like receptor (NLR) containing purine domain-3 proven in chronic obstructive pulmonary disease and IPF. In addition, IL-1β enhances the IL-6 and TNF-α expression, disrupting the alveolar architecture, leading to collagen deposition and increased lung fibroblasts [7,92].

In this study, we reported that significantly elevated IL-1β, IL-6, and TNF-α levels, as well as collagen synthesis, in BLM-treated lung tissues and in mouse plasma were reduced by the *L. minor* addition i.e., 120 mg/mL extract induces cell proliferation of lung fibroblasts. It should be noted that in this report we showed the 33-day induction of a probable anti-inflammatory therapeutic effect of L. minor in bleomycin-induced pulmonary fibrosis models in both tissue and blood in addition to the 16-day preventive effect. The results suggest BLM-induced inflammation inhibition of *L. minor* extract and decreased caused ROS influx through regulation of IL-1β, IL-6, and TNF-α balance, after 16 days and 33 days course. In agreement, lemnan, *L. minor* isolated apiogalacturonanic pectin regulates the secretion of Th2-type IgG subclass after 28 days and after stimulation of a transient IL-4 burst suggests restorative IL-4 expression [68]. Further, Kalmakhelidze, ref. [93] emphasizes that *L. minor* orally administrated mice showed a regenerative process in the small intestine lining after 5 Gy irradiation and probably regenerated IL-1β, IL-6, and TNF-α levels. Also, *L. minor* extracts (>233 ng/mL) do not cause cell necrosis and partially prevent apoptosis on CD_4_^+^ cells, CD_8_^+^ cells and B lymphocytes within 48 h [58]. Other experimental research [40], revealed that PPs, isolated from duckweed/*Lemna* species stimulated immunoregulatory mediators such as (IL)-1β, IL-10, TNF-α, nitric oxide (NO), ROS and produce related pro-inflammatory immune responses. In addition, PPs-HG domain considered to be the preferential domain for alleviating acute colorectal inflammation [39,40].

In a compositional ratio containing phytosterols, hydrocarbons, aldehydes, ketones, lipids and amino acids and proteins (23.97%), *L. minor* extract has the potential to inhibit and neutralize oxyl and peroxyl radicals [94], which are directly related with lipid peroxidation levels. In addition, Gülçin et al. [95] confirmed the *L. minor* aqueous extract antioxidant capacity compared to α-tocopherol (84.6%) and Trolox (95.6%) and the possibility of maximal lipid peroxidation inhibition in vitro, even at a concentration (45μg/mL) four times lower than we used. In vitro and in vivo, xenobiotic phytotoxic doses of BLM, vincristine, ciprofloxacin have been found to initially impair the antioxidant enzyme *L. minor* activity, attributable to ROS-induced irreversible oxidative proteolytic O_2_^•−^ non-regulation [5,34,96]. Therapeutic *L. minor* use as an antioxidant substance [96] refutes these facts and can be an important resource in neutralizing oxidative damage and ROS indication in inflammatory infiltrates and IPF caused by BLM [25]. In line with our results, long-term L. minor use as an antioxidant food source with a high amino acids and proteins content increases body weight, improves physical data and maximally activates pulmonary antioxidant protection [25,97,98].

The effect of combination therapy with *L. minor* extract on the levels of oxidized proteins, glycation end products, antioxidant enzymes and anti-inflammatory cytokines in BLM-induced inflammation and fibroticity has never been reported in the literature; therefore, no statistical comparison of the results of the present study can be made. However, the results of the present study highlight the need for further research to determine whether a higher dose of *L. minor* induced orally, intrapetitonially, etc., or included as a dietary supplement may be advantageous in terms of the studied parameters. The stable reduction of protein carbonylation, lipid peroxidation, ROS deactivation and cytokine modulation of 120 mg/mL *L. minor* used in the present study showed beneficial protective effects on both antioxidant protection and anti-inflammatory activity of the toxin-induced without causing toxicity on its own. Further studies are needed to investigate the use of *L. minor* as a protector in other pathological conditions, in diets, in the vaccines production.

## 5. Conclusions

In conclusion, our study shows that chronic BLM exposure leads to irreversible changes in lung function and obvious histological fibrotic delay. The use for the first time of L. minor as an antioxidant source and cyclic re-modulator improves the protective response of membrane proteins, carbonyl stress and reduced OS disorders during the inflammatory process and the IPF initiation. Our study demonstrates that MCs appear to be critical to pulmonary fibrosis. According to Veerappan et al. [98], the therapeutic blockade of mast cell degranulation and/or histamine and ANG II receptors should attenuate pulmonary fibrosis. Our data suggest that the *L. minor* ability to impair MCs survival and activation may be a novel and additional mechanism by which *L. minor* exerts its anti-fibrotic effects in patients with IPF.

## Figures and Tables

**Table 1:** BLM-induced lung changes caused body weight (BW) loss (g).

Administration	BW (g)	BW (g)	BW (g)	BW (g)
(*n* = 6)	5th day	9th day	16th day	33rd day
BLM	189.6 ± 2.44 *	173.4 ± 1.22 *	155.5 ± 2.9 *	145.9 ± 3.09 *
*L. minor*	203.2 ± 1.4	211 ± 3.06	223.8 ± 2.05	251.3 ± 2.05
BLM + *L. minor*	205.1 ± 1.91	213.8 ± 2.71 *	221.9 ± 1.6 *	241.8 ± 2.07 *
controls	207.2 ± 3.03	221.09 ± 1.92	230.5 ± 2.84	258.5 ± 3.06

The body weights of experimental animals were measured at 5th, 9th, 16th and 33rd days during the experimental periods in four groups: controls (standart diet, *n* = 6); BLM (*n* = 6); *L. minor* (*n* = 6); BLM + *L. minor* in combination (*n* = 6). The quantitative data were expressed as the means ± SE. * *p* < 0.05 compared with control.

**Table 2:** *L. minor* effect on BLM-induced alterations in lung (count metachromatic MCs) and airway and blood vessels histology of BALB/c mice.

Parameters	Controls	BLM	*L. minor* + BLM	*p*
MCs Number				
Large bronchi wall	5.40 ± 0.55 (A4/B2)	16.20 ± 4.76 (C4)	9.40 ± 0.55	*p* ˂ 0.005
Small bronchi wall	- (A3/B3)	4.20 ± 0.83	4.00 ± 1.00	*p* ˂ 0.01
Interalveolar septa	0.40 ± 0.55 (A3)	5.20 ± 0.84 (C1)	1.60 ± 0.55	*p* ˂ 0.001
Blood vessel adventitia	1.40 ± 0.55 (A1)	5.20 ±1.09	4.60 ± 1.14	*p* ˂ 0.0001
Interalveolar septa thickness (µm)	7.45 ± 1.60 (A4/C4)	14.74 ± 3.05	8.39 ± 1.31	*p* < 0.001

Mast cell (MCs) number per a microscopic field is given as mean ± standard deviation (SD) in the wall of bronchi and blood vessels, and in the interalveolar septa in the controls, the BLM group, and BLM + L. minor treated mice on day 33. Interalveolar septa were thicker in BLM treated group than in BLM + *L. minor* group and controls (*p* < 0.001). Legend: —absence of mast cells; A—statistical significant difference between controls and BLM group; B—statistical significant difference between controls and BLM + L. minor group; C—statistical significant difference between BLM and BLM + *L. minor* groups; 1, 2, 3, 4 express the values of *p* (*p* ˂ 0.005, *p* ˂ 0.01, *p* ˂ 0.001, *p* ˂ 0.0001, respectively).

## Data Availability

All of the data is contained within the article.
